# Screening‐based identification of xanthone as a novel NLRP3 inflammasome inhibitor via metabolic reprogramming

**DOI:** 10.1002/ctm2.496

**Published:** 2021-07-19

**Authors:** Wenyu Cui, Sheng Chen, Zhexu Chi, Xingchen Guo, Xiaobo Zhang, Yueyang Zhong, Haijie Han, Ke Yao

**Affiliations:** ^1^ Eye Center of the 2nd Affiliated Hospital Zhejiang University School of Medicine Hangzhou Zhejiang Province China; ^2^ Zhejiang Provincial Key Lab of Ophthalmology Hangzhou Zhejiang Province China; ^3^ Institute of Immunology Zhejiang University School of Medicine Hangzhou Zhejiang China; ^4^ Department of Colorectal Surgery and Oncology Key Laboratory of Cancer Prevention and Intervention, Ministry of Education The Second Affiliated Hospital, Zhejiang University School of Medicine Hangzhou Zhejiang Province China; ^5^ Cancer Center Zhejiang University Hangzhou Zhejiang Province China; ^6^ State Key Laboratory of Virology College of Life Sciences Wuhan University Wuhan Hubei Province China

Dear Editor,

The NLRP3 inflammasome is an intracellular surveillance multimolecule platform that senses broad ranges of pathogen‐derived, environmental, and endogenous stress‐induced factors,^1^ and aberrant NLRP3 inflammasome activation is associated with multiple inflammation‐related disorders and diseases, such as gout, T2D, and atherosclerosis.^2^ Since then, newly discovered NLRP3 inflammasome inhibitor may serve as a lead for developing new therapeutics against these diseases. In this study, we discovered a novel small‐molecular natural product, xanthone, which could inhibit NLRP3 inflammasome activation both *in vivo* and *in vitro* by configuring cellular metabolic profile.

In order to discover novel NLRP3 inflammasome inhibitors, we treated primary macrophages with various natural products upon NLRP3 inflammasome activation. Out of more than 40 compounds tested, we determined xanthone as a potential target (Figures 1A and 1B). We then showed xanthone dose‐dependently inhibited NLRP3 inflammasome activation, but not its priming step, nor directly influencing cell pyroptosis after inflammasome assembly (Figures 1C and 1D and Figure S1A–S1G). Furthermore, xanthone could attenuate a broad range of stimuli‐induced NLRP3 inflammasome activation (Figures 1E and 1F and Figure S1H), and even pan‐inflammasome activation (Figures S1I and S1J). Xanthone treatment could also inhibit the cleavage of Gsdmd (Figures S1K and S1L). And we showed xanthone could significantly restrain IL‐1β production of human peripheral blood mononuclear cells (Figure S2A). One step further, we found xanthone could inhibit *in situ* ASC speck formation both in primary and ASC‐GFP expressing immortalized macrophages. (Figure 1G and Figures S2B and S2C). Consistently, xanthone could significantly inhibit cross‐link ASC oligomerization and endogenous NLRP3 oligomerization *in vitro* (Figures 1H and 1I), without affecting NLRP3 inflammasome components protein level (Figure 2D).

**FIGURE 1:**
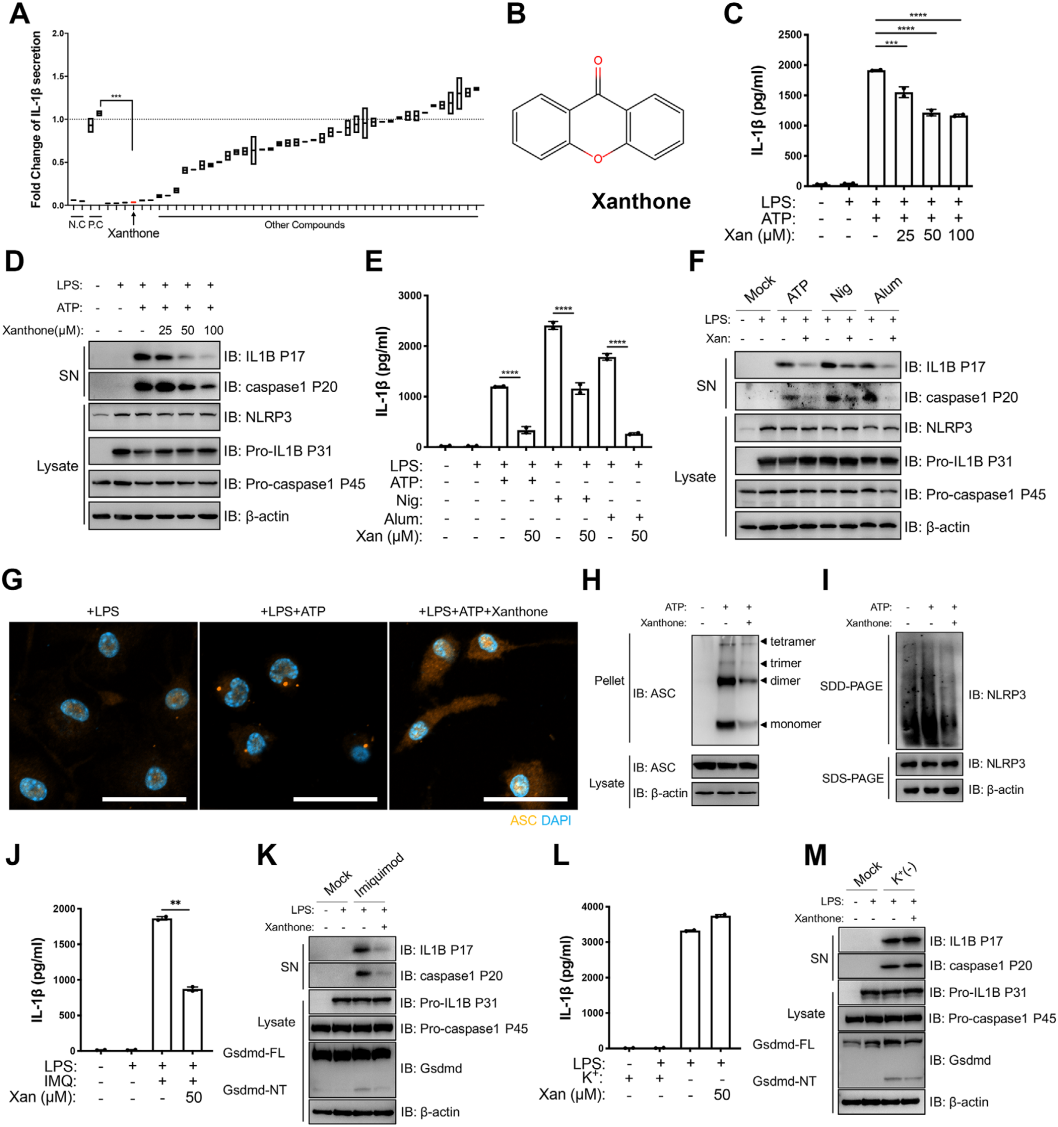
Xanthone dose‐dependently inhibits NLRP3 inflammasome activation with no effect on priming step. (A) Unbiased screening of natural NLRP3 inhibitor using LPS‐primed peritoneal macrophages. IL‐1β level of LPS+ATP group (positive control, P.C) was set as 1.0. (B) Structure of xanthone. (C and D) LPS‐primed peritoneal macrophages treated with different doses of xanthone 2 h before ATP challenge. Supernatants (SN) and cell extracts (lysate) were analyzed by immunoblotting (D), IL‐1β (C) secretion was determined by ELISA. (E and F) LPS‐primed peritoneal macrophages treated with 50 μM xanthone for 2 h, followed by stimulation with ATP, Nigericin (N), aluminum salts (Alum). Supernatants (SN) and cell extracts (Lysate) were analyzed by immunoblotting (F). Supernatants were also analyzed by ELISA for IL‐1β (E). (G and H) Representative immunofluorescence images of ASC speck formation of LPS‐primed BMDMs with indicated treatment (G), and ASC oligomerization in cross‐linked cytosolic pellets analyzed by immunoblotting in (H). Scale bars, 20 μm. (I) Semi‐denaturing detergent agarose gel electrophoresis (SDD‐AGE) detection of NLRP3 oligomerization. (J–M) LPS‐primed peritoneal macrophages treated with 50 μM xanthone before 100 μM Imiquimod challenge (J and K) or substitution of K^+^‐free medium challenge for 30 min (L and M). Supernatants (SN) and cell extracts (Lysate) were analyzed by immunoblotting (K and M), IL‐1β (J and L) secretion was determined by ELISA. *p < 0.05, **p < 0.01, ***p < 0.001, ****p < 0.0001, two‐tailed unpaired Student's *t*‐test. Data are the mean ± SD

**FIGURE 2:**
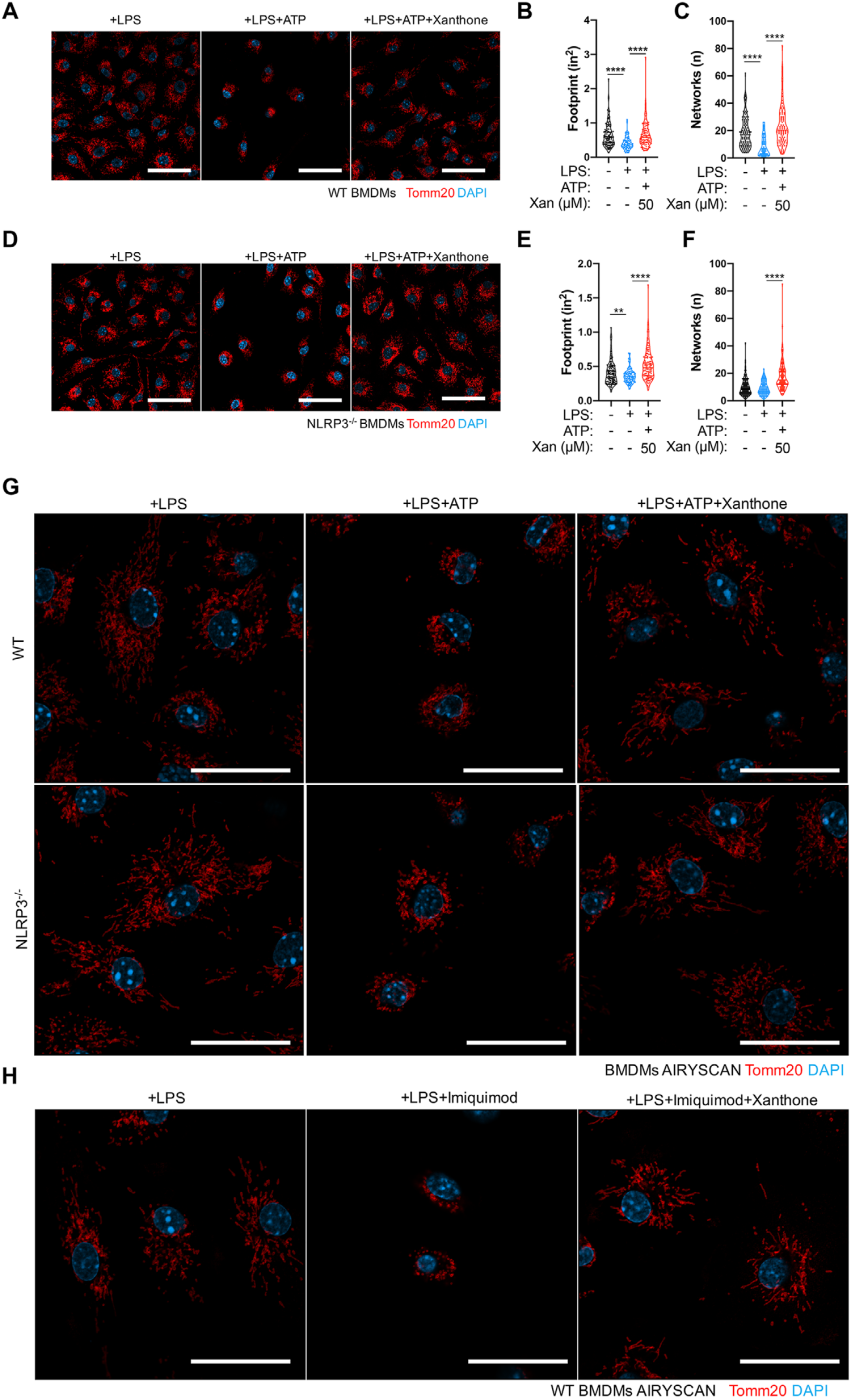
Xanthone mitigates mitochondrial dysfunction upon NLRP3 inflammasome activation. (A–F) Representative confocal images of LPS‐primed WT (A–C) or NLRP3 KO (D–F) BMDMs pretreated with xanthone or not after ATP stimulation for 30 min (A and D) and statistics of mitochondrial area (footprint, B and E) and networks (C and F) with MiNA. Scale bars, 50 μm. (G and H) Super‐resolution images of LPS‐primed WT (G, upper panel) or NLRP3 KO (G, lower panel) BMDMs pretreated with xanthone or not after ATP or imiquimod (H) stimulation for 30 min, using Airyscan mode. Scale bars, 20 μm. **p < 0.01, ****p < 0.0001, one‐way ANOVA

Mitochondria dysfunction and potassium efflux are two most common triggers of NLRP3 inflammasome activations.^1^, ^3^ We showed that xanthone could significantly inhibit mitochondrial damage specific, but not potassium efflux‐induced NLRP3 activation (Figures 1J–1M and Figures S2E and S2F). NLRP3 inflammasome activation is strictly regulated by altered mitochondrial fitness and dynamics,^3^, ^4^ and we found that xanthone could prevent ATP‐induced mitochondria fission, area loss and network disruption (Figures 2A–2C). Moreover, xanthone could prevent mitochondrial fission in NLRP3 KO macrophages (Figures 2D–2F), indicating its effect was at the early burst rather the later inflammasome‐mediated amplification stage of mitochondrial damage.^5^ Using high‐resolution microscopy, we also visualized that xanthone could prevent mitochondria fragmentation, indicating xanthone could prevent the disturbance of mitochondrial integration upon NLRP3 activation (Figures 2G and 2H).

Being the metabolic hub of cell, mitochondria function and dynamics are closely related to cellular metabolic profile.^6^, ^7^ In particular, NLRP3 inflammasome activation is precisely regulated by metabolic pathways, such as glycolysis, fatty acid oxidation, and so on.^2^ Since then, we first detected cell oxygen consumption rate (OCR) upon ATP stimulation (Figure 3A) and determined that xanthone treatment could prevent NLRP3 inflammasome activation‐induced drop of cell maximal respiration (Figures 3A and 3B). Besides, xanthone could significantly rescue the reduction of mitochondrial spare capacity and proton leak (Figures 3C and 3D and Figure S3A), with little effect on nonmitochondrial respiration (Figure S3B). Accompanied with the attenuation of metabolic disruption, we found that xanthone could elevate mitochondrial membrane potential and decrease mtROS generation (Figure 3E). These data indicated that xanthone could restore macrophage mitochondrial fitness upon NLRP3 activation.

**FIGURE 3:**
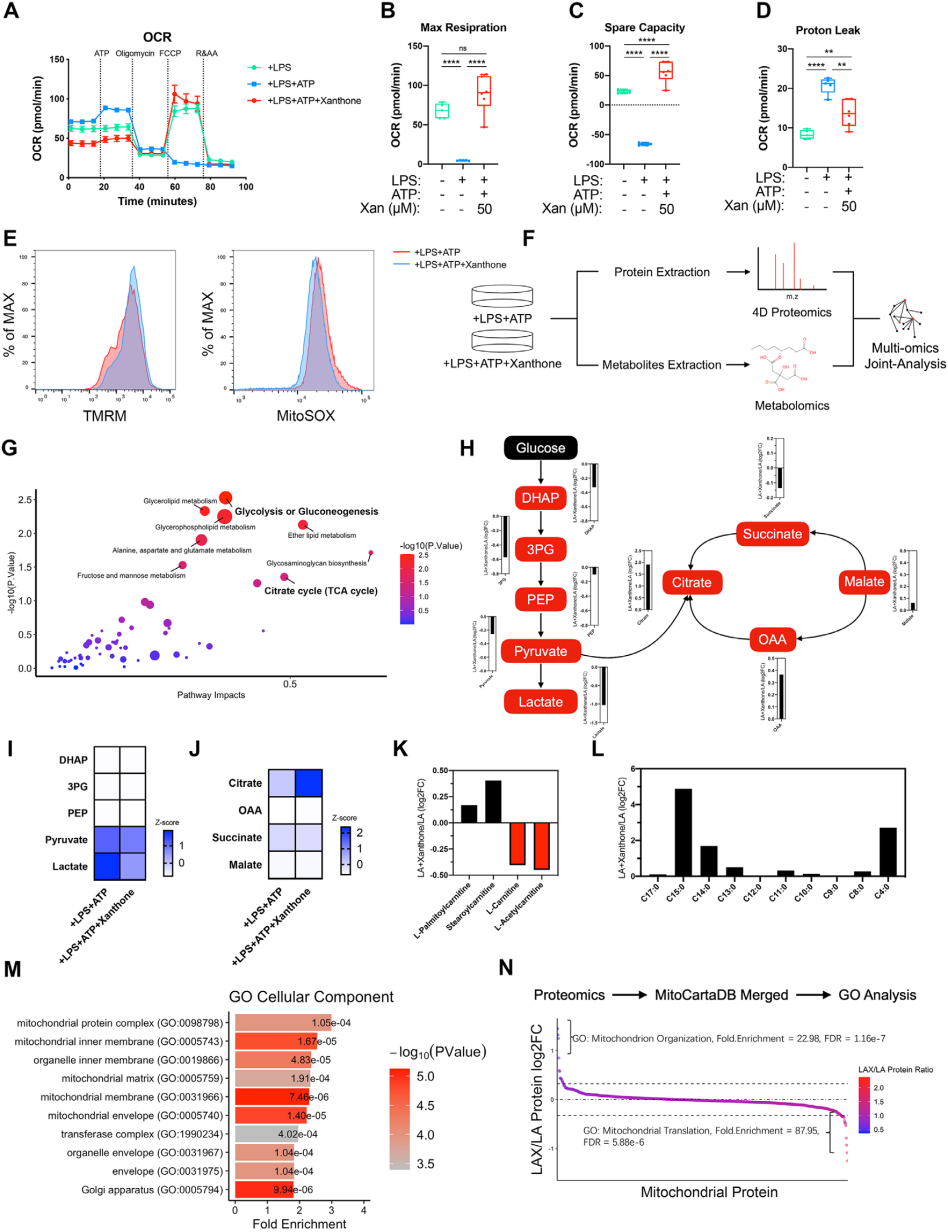
Xanthone metabolically rewires macrophages to regain mitochondrial fitness upon NLRP3 inflammasome activation. (A–D) Cell oxygen consumption rate (OCR) with the indicated treatments. LPS‐primed BMDMs were treated with xanthone and VX765 (10 mM) and then loaded, followed by sequential treatment with ATP, Oligo (1 mM), fluorcarbonyl cyanide phenylhydrazone (FCCP; 1 mM), and rotenone and antimycin A (R&AA, 1 and 5 mM, respectively), as indicated (A). Maximal respiration (B), spare capacity (C), and proton leak (D) were calculated with Wave 2.4.0. (E) Mitochondrial ROS and mitochondrial membrane potential were measured by MitoSOX incorporation (left panel) and TMRM (right panel) using flow cytometry. (F and G) Workflow of extraction and joint‐analysis of 4D unlabeled proteomics and untargeted metabolomics (F). Joint analysis of 4D unlabeled proteomics and untargeted metabolomics data using MetaboAnalyst 5.0 (G). (H–J) Fold changes of glycolysis (I) and TCA cycle (J) intermediates after xanthone treatment compared with control macrophages. (K and L) Fold changes of fatty acid metabolism intermediates. (M) GO cellular component enrichment of total differentiated proteins. (N) Proteomics data was merged with MitoCartaDB followed by GO enrichment analysis with protein ratio changed larger than 1.25 fold. **p < 0.01, ****p < 0.0001, one‐way ANOVA

In order to investigate how xanthone regain mitochondrial integration, we employed both 4D proteomics and untargeted metabolomics analysis (Figure 3F). We illustrated that xanthone treatment could lead to exceeding changes of glucose metabolism (Figure 3G), evidenced by a dramatic shift from glycolysis toward citrate cycle, with trivial changes of enzymes protein level (Figures 3H–3J and Figures S3C and S3D). This shift of intermediate metabolites level was consistent with restricted inflammasome activation.^8^, ^9^ To demonstrate the metabolic clues behind, we further discovered that xanthone could induce a conversion of free‐carnitines toward fatty acid‐conjugated carnitines and more medium‐/short‐free fatty acids, indicating an elevation of carnitine‐shuttle capacity and fatty acid oxidation (Figures 3K and 3L). The most enriched GO cellular component terms were mitochondrial protein complex and inner membrane (Figure 3M), and xanthone could significantly upregulated cellular response to stress (Figure S3E). To further depict the full spectrum of mitochondria integration, we merged our data with MitoCartaDB before enrichment (Figure 3N). Xanthone treatment elevated mitochondrial organization, while control cells were enriched with mitochondrial translation, representing the response toward dysfunction.^10^ Next, we found complex I inhibitors rotenone and piericidin A could dose‐dependently elevate IL‐1β production in xanthone‐treated macrophage, with trivial effect on control cells at such low concentration (Figures S4A–S4C). And rotenone could restore xanthone‐prevented mitochondria fission (Figure S4D). Taken together, we illustrated that xanthone metabolically rewired macrophage, which regained its mitochondria fitness and limited NLRP3 inflammasome activation.

We finally asked whether xanthone could suppress NLRP3 inflammasome *in vivo*. Intraperitoneal injection of xanthone could significantly decrease sera IL‐1β level, improve survival rate, with no change of TNFα level in LPS‐induced sepsis mice (Figures 4A–4C). In addition to systemic sepsis, intrastromal injection of LPS is sufficient to trigger excessive local inflammation response. We found that xanthone delivery significantly dampened LPS‐mediated keratitis, with less corneal opacification and clearer cornea performance (Figures 4D and 4E). Xanthone treatment lowered corneal infiltrate density and decreased corneal thickness (Figures 4F and 4G), without affecting cornea epithelial cells viability (Figure S5A). Furthermore, xanthone could inhibit LPS‐induced IL‐1β secretion, pro‐caspase‐1 maturation as well as Gsdmd cleavage, and significantly inhibit FLICA intensity in tissue‐infiltrating macrophage, representing less mature caspase‐1 activity (Figures 4H–4L and S5B).

**FIGURE 4:**
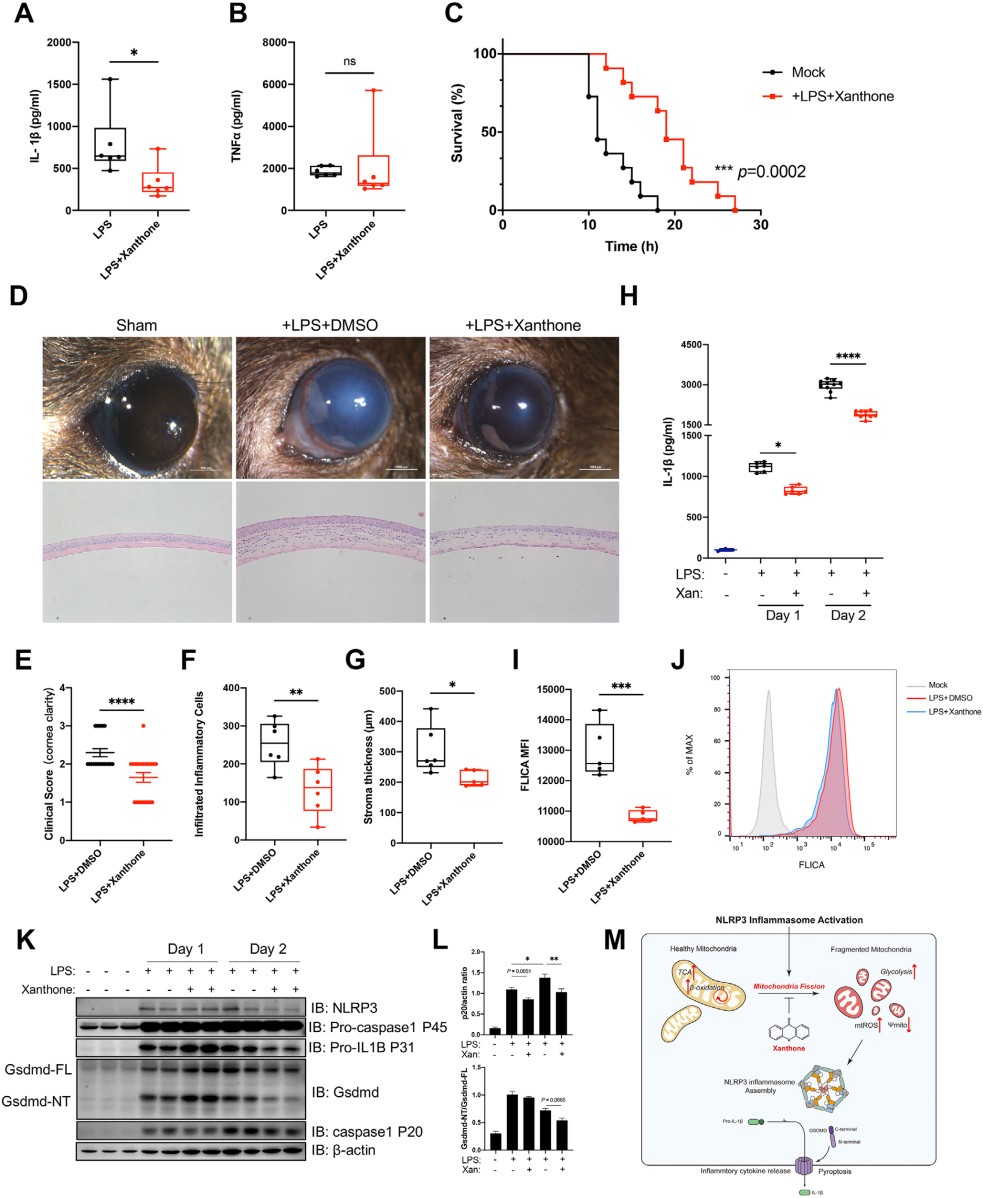
Xanthone treatment ameliorates LPS‐induced sepsis and mice keratitis. (A and B) ELISA of IL‐1β (A) and TNFα (B) in sera from mice intraperitoneally injected with saline or xanthone (20 mg/kg body weight) with LPS (25 mg/kg body weight) for 4 h. Data are the mean ± SEM (*n* = 6 mice/group). (C) Survival of mice intraperitoneally injected with saline or xanthone (20 mg/kg body weight) with LPS (20 mg/kg body weight, *n* = 11 mice/group). (D–G) Mice were sham operated or intrastromal injected with LPS and treated with xanthone or DMSO control three times a day for 24 or 48 h. Representive clinical images were taken with stereomicroscope (D, upper panel), and H&E staining of mice corneal (D, lower panel). Mice corneal clinical scoring was based on a 4‐point system (E). Average infiltrated inflammatory cells number was counted at least three slides manually (F), while stroma thickness was determined by ImageJ (G). (H) Intrastromal IL‐1β level was determined by ELISA 24 and 48 h after LPS injection. (I and J) The level of NLRP3 inflammasome activation level of mice corneal infiltrated macrophage was determined with FLICA staining (I), and representative histogram was shown (J). (K and L) Lysates of mice cornea was determined by Western blot (K) and quantifications were normalized determined by ImageJ (L). (M) Schematic representation of the inhibition effect of xanthone upon NLRP3 inflammasome activation. *p < 0.05, **p < 0.01, ***p < 0.001, ****p < 0.0001, two‐tailed unpaired Student's *t*‐test and one‐way ANOVA, Log‐rank (Mantel–Cox) test for survival

In summary, based on unbiased screening, we newly identified xanthone as a NLRP3 inflammasome inhibitor. Mechanistically, we showed xanthone metabolically rewired macrophages to obtain mitochondria fitness upon NLRP3 inflammasome activation (Figure 4M). Our *in vivo* data showed the beneficial effects of xanthone in both LPS‐induced systemic sepsis and topical keratitis. Together, our data showed that xanthone could serve as a promising inhibitor to treat excessive NLRP3‐related diseases.

## CONFLICT OF INTEREST

The authors declare no conflict of interests.

## Supporting information

Supporting Information
