# Supplementary material for: Screening‐based identification of xanthone as a novel NLRP3 inflammasome inhibitor via metabolic reprogramming
Source: Clin Transl Med. 2021 Jul 19;11(7):e496. doi: 10.1002/ctm2.496 (PMC8288006; doi:10.1002/ctm2.496)
Supplement: Supplementary file 1 — Supporting Information [file CTM2-11-e496-s001.docx]

**Supplementary Materials for**

**Screening based identification of xanthone as a novel NLRP3 inflammasome inhibitor via metabolic reprogramming**

Wenyu Cui^1,2,7^, Sheng Chen^3,4,5,7^, Zhexu Chi^3^, Xingchen Guo^6^, Xiaobo Zhang^1,2^, Yueyang Zhong^1,2^, Haijie Han^1,2^, Ke Yao^1,2*^

^1^Eye Center of the 2nd Affiliated Hospital, Zhejiang University School of Medicine, Hangzhou, Zhejiang Province, China

^2^Zhejiang Provincial Key Lab of Ophthalmology, Hangzhou, Zhejiang Province, China

^3^Institute of Immunology, and Department of Orthopaedic Surgery of the Sir Run Run Shaw Hospital

^4^Department of Colorectal Surgery, the Second Affiliated Hospital of Zhejiang University School of Medicine.

^5^Cancer Institute (Key Laboratory of Cancer Prevention and Intervention, China National Ministry of Education; Key Laboratory of Molecular Biology in Medical Sciences, Zhejiang Province, China), the Second Affiliated Hospital, Zhejiang University School of Medicine.

^6^State Key Laboratory of Virology, College of Life Sciences, Wuhan University, Wuhan 430072, Hubei Province, China

^7^These authors contributed equally to this work

*Correspondence: xlren@zju.edu.cn (K. Yao)

**This PDF file includes:**

Methods and Materials, Figures S1 to S5

**1** | **MATERIALS AND METHODS**

**1.1** | **Cells**

Mouse peritoneal macrophages were harvested 4 days after thioglycolate (Merck) injection. Mouse bone marrow cells were flushed from tibias and femurs with chilled Dulbecco’s modified Eagle’s medium (DMEM) and then cultured in DMEM supplemented with 10% fetal bovine serum (FBS), 1% penicillin/streptomycin, and 10 ng/ml macrophage colony-stimulating factor (PeproTech) to generate BMDMs. Construction of ASC-GFP-iBMDMs were described previously ^12^. Fresh human PBMCs were isolated from healthy volunteers from Zhejiang University.

Stimulation conditions were as described previously ^27^. Briefly, cells were primed with LPS (500 ng/mL) for 4 h in Opti-MEM, and followed by stimulation with various NLRP3 inflammasome activators [2 mM ATP (30 min); 10 µM nigericin (30 min); 300 mg/mL aluminum salts (5 h)]. Cells were pretreated with irreversible caspase-1 inhibitor VX765 (10mM) 30 min before ATP or other 2nd stimuli challenge in order to minimize the effects of different levels of pyroptosis for imaging, omics, flow cytometry, as well metabolic assays, etc. experiments. Cell lysates and precipitated supernatants were further analyzed by western blotting and ELISA.

**1.2** | **Mice**

C57BL/6 mice were purchased from the Model Animal Research Center of Nanjing University. *Nlrp3*^-/-^ C57BL/6 mice were from The Jackson Laboratory. All mice were housed in a specific pathogen-free facility in the Laboratory Animal Center of Zhejiang University in accordance with the National Institute of Health Guide for Care and Use of Laboratory Animals. The animal experimental protocols were approved by the Review Committee of Zhejiang University School of Medicine and were in compliance with institutional guidelines.

**1.3** | **Quantitative PCR (qPCR)**

RNA was extracted using total RNA extraction reagent (Vazyme Biotech). Complementary DNA was synthesized using HiScript® II Reverse Transcriptase (Vazyme Biotech) according to the manufacturer’s instructions. QPCR was performed using SYBR Green (Vazyme Biotech) on a CFX96 Touch Real Time PCR (BioRad). All samples were individually normalized to GAPDH. The following primers were used, *Nlrp3*: FP 5’-TGCTCTTCACTGCTATCAAGCCCT-3’, RP 5’-ACAAGCCTTTGCTCCAGACCCTAT-3’, *Il1β*: FP 5’-TGGAAAAGCGGTTTGTCT-3’, RP 5’-ATAAATAGGTAAGTGGTTGCC-3’, *Il18*: FR 5’-TGGTTCCATGCTTTCTGGACTCCT-3’, RP 5’-TTCCTGGGCCAAGAGGAAGTGATT-3’, *iNOS*: FP 5’- CAGGGAGAACAGTACATGAACAC-3’, RP 5’- TTGGATACACTGCTACAGGGA-3’.

**1.4** | **Reagents and antibodies**

ATP (A7699), LPS (L2630), gramicidin (368020), PMA (P1585), Rotenone (R8875) were from Sigma; Nigericin (tlrl-nig) and poly (dA:dT) (tlrl-patn) were from Invivogen; VX-765 and natural product library were from Selleck chemicals; imiquimod (HY-B0180), xanthone (HY-N0126) were from MedChemExpress; Imject^TM^ Alum Adjuvant (77161) was from Thermo Fisher; Antimycin A (ab141904) was from Abcam; Piericidin A (C3535) was from APExBIO. Image-iT TMRM Reagent (I34361), MitoSOX Red (M36008) were from Thermo Fisher; and *Salmonella* was a kind gift from Prof. Yongqun Zhu of Zhejiang University.

Anti-IL-1b (Cat# AF-401-NA, RRID: AB_416684) was from R&D Systems, anti-Caspase-1 (Cat# AG-20B-0042, RRID: AB_2490248), anti-ASC (Cat# AG-25B-0006, RRID: AB_2490440), anti-NLRP3 (Cat# AG-20B-0014, RRID: AB_2490202) were from Adipogen. Anti-GSDMD (ab209845) was from Abcam. Anti-β-actin (Cat# db10001) was from Daigebio and anti-Tomm20 (Cat# ET1609-25) was from HuaAn biotechnology. Flow cytometry antibodies PE-Cy7-CD11b, APC-Cy7-CD45, APC-F4/80 were from Biolegend. Fc blocker (Cat# 553141, RRID: AB_394656) was from BD PharMingen.

**1.5** | **Immunoblot analysis**

Cells were lysed in 2×SDS loading buffer [100 mM Tris-HCl, 4% SDS, 20% glycerol, 2% 2-mercaptoethanol, and 0.05% bromophenol blue]. The samples were then boiled and separated on 10% or 12% SDS-PAGE gels and transferred to nitrocellulose membranes (Pall, #28637358). The membranes were blocked for 1 h in blocking buffer (5% skimmed milk and 0.1% Tween 20 in TBS) at room temperature. After incubation with primary and secondary antibodies, ECL blotting reagents (Thermo Fisher) were used for immunoblot detection.

**1.6** | **Mitochondrial ROS and membrane potential detection**

Mitochondrial ROS was measured by incubation with 5 mM MitoSOX or 50 nM tetramethylrhodamine-methyl ester-perchlorate (TMRM) in PBS for 30 min at 37°C (Thermo Fisher Scientific). The cells were rinsed in cold PBS and analyzed using LSR Fortessa (BD Biosciences) or NovoCyte (ACEA Bioscience Inc.) flow cytometers. Data were processed using FlowJo software (FlowJo v10).

**1.7** | **Metabolic assays**

The oxygen consumption rate (OCR) was measured using a 96 well Extracellular Flux Analyzer (Seahorse, Agilent) according to the manufacturer’s instructions. Briefly, 1*10^5^ BMDMs/well were cultured overnight in Seahorse XFe96 cell culture microplates. The following day, the cells were pre-incubated at 37°C for a minimum of 45 minutes in the absence of CO2 in DMEM (Seahorse, Agilent) with 25 mM glucose, 1 mM pyruvate and 2 mM GlutaMAX (GIBCO) with pH adjusted to 7.4. The OCR were measured with/without the following reagents: 2 mM ATP, 1 mM oligomycin, 1 mM flurorcarbonyl cyanide phenylhydrazone (FCCP), and 1 mM rotenone + 5 mM antimycin A (Abacm) as indicated. All results were analyzed with Wave software version 2.4.0 (Agilent).

**1.8** | **ASC oligomerization and ASC speck formation**

ASC oligomerization and speck formation conditions were as previously described ^27^. Briefly, after stimulation, cells were rinsed in PBS and 500 µL ice-cold buffer [50 mM Tris-HCl pH 7.6, 0.5% Triton X-100, 0.1 mM PMSF, and protease inhibitor cocktail] was added. Cells were scraped, lysed, and centrifuged at 330 g for 10 min and the pellets were washed twice and re-suspended in 500 µL PBS. Disuccinimidyl suberate (2 mM) was added to the re-suspended pellets, and then incubated at room temperature for 30 min with rotation. Samples were then centrifuged and re-suspended in SDS loading buffer for western blotting.

For ASC speck formation, ASC-GFP-iBMDMs were seeded and cultured overnight on glass coverslips. The following day, the cells were primed with LPS and treated with ATP in the presence or absence of the indicated inhibitors. The cells were fixed in 4% paraformaldehyde followed by detection.

**1.9** | **NLRP3 oligomerization**

The oligomerization of NLRP3 was analyzed according to the published protocol ^23^. Briefly, cells were lysed with Triton X-100 lysis buffer (0.5% Triton X-100, 50 mM Tris–HCl, 150 mM NaCl, 10% glycerol, 1 mM PMSF, and protease inhibitor cocktail), and then resuspended in 1× sample buffer (0.5× TBE, 10% glycerol, 2% SDS, and 0.0025% bromophenol blue) and loaded onto a vertical 1.5% agarose gel. After electrophoresis in the running buffer (1× TBE: 89 mM Tris (pH 8.3), 89 mM boric acid, 2 mM EDTA and 0.1% SDS) for 1 h with a constant voltage of 80 V at 4°C, followed by immunoblotting.

**1.10** | **ELISA**

Supernatants from cell cultures and sera were collected and the concentrations of IL-1β, IL-18, and TNFα (all from ThermoFisher) were determined according to the manufacturer’s instructions.

**1.11** | **Immunofluorescence staining and confocal microscopy**

BMDMs were cultured overnight on glass coverslips. After stimulation, the cells were then fixed with 4% paraformaldehyde in PBS for 15 min, and permeabilized with 0.2% Triton X-100. After blocking with 5% BSA (Solarbio), cells were incubated with primary antibodies overnight at 4°C in PBS containing 1% BSA. The following day, after three times washes in PBS with Tween 20 (PBST), the cells were incubated with secondary antibodies in PBS for 1 h at room temperature and rinsed in PBST, then mounted with mounting media containing DAPI (Sigma). Airyscan model was used for super-resolution image gaining. Analyses were carried out using a Zeiss LSM880/800 microscope. Mitochondrial footprint and networks were analyzed by image J with MiNA macro.

**1.12** | **Cellular viability assay**

The cellular viability was evaluated using Cell Counting Kit-8 (CCK-8; beyotime, China) according to the manufacturer's instructions. The cultured corneal epithelial cells were cultivated in a 96-well plate (5 × 10^3^ cells in 100 µL /well) for 24 hours, then treated with different concentrations of Xanthone. After treatment of 24 hours, CCK-8 reagent (10 µL) was added to each well. Cells were further incubated for 1 hour. The absorbance at 450 nm was measured using microplate reader.

**1.13** | ***In vivo* LPS challenge**

C57B6 mice were injected intraperitoneally with LPS. For the sepsis model, mice were sacrificed 4 h after LPS challenge (25 mg/kg body weight), and the serum levels of IL-1β, IL-18, and TNFα were measured by ELISA (Thermo Fisher) according to the manufacturer’s instructions. For the survival model, mice were challenged with LPS (20 mg/kg body weight) and observed for up to 30 h.

**1.14** | **LPS-induced keratitis**

C57B6 mice were intrastromal injected with 2µL of 1mg/mL LPS with one eye, and PBS for the remaining eye. Xanthone or DMSO control (all diluted in PBS) was topically delivered three times a day. Mice corneas were collected 24 or 48 hours later for clinical scoring, H&E staining, western blot or flow cytometry.

For clinical scoring, a 4-point system was used based on published protocol ^28^. Briefly, 0 = normal, clear cornea; 1 = mild corneal haze, but still able to see iris structures; 2 = moderate corneal haze, difficult to see iris structures; 3 = Significant corneal opacity, unable to see iris structures; 4 = damage and loss of corneal tissue (including melt).

For flow cytometry of mice corneal infiltrating macrophages, corneas were digested with collagenase I (Sigma) and filtered with 70 µm filters. Cells were Fc blocked and stained with indicated antibodies and FAM-FLICA (Immunochemistry) for 30 min on ice, followed by flow cytometry detection.

For western blot, mice corneas were lysed in RIPA (with 1 mM PMSF, and protease inhibitor cocktail) on ice for 2 hours. The lysate was mixed with loading buffer and detected by western blot.

**1.15** | **Untargeted metabolomics**

Macrophages were stimulated as indicated and collected and untargeted metabolomics was done with the assistant of BioTree, Shanghai. Briefly, after extraction, LC-MS/MS analyses were performed using an UHPLC system (Vanquish, Thermo Fisher Scientific) with a UPLC BEH Amide column (2.1 mm × 100 mm, 1.7 μm) coupled to Q Exactive HFX mass spectrometer (Orbitrap MS, Thermo). The raw data were converted to the mzXML format using ProteoWizard and processed with an in-house program, which was developed using R based on XCMS, for peak detection, extraction, alignment, and integration. Then an in-house MS2 database (BiotreeDB) was applied in metabolite annotation. The cutoff for annotation was set at 0.3.

**1.16** | **4D label-free proteomics**

4D label-free proteomics was performed with the assistance of PTM Bio (Hangzhou). Briefly, after indicated stimulation, cells were collected and sonicated three times on ice using a high intensity ultrasonic processor (Scientz) in lysis buffer (8 M urea, 1% Protease Inhibitor Cocktail). The remaining debris was removed by centrifugation at 12,000 g at 4 °C for 10 min. Finally, the supernatant was collected and the protein concentration was determined with BCA kit according to the manufacturer’s instructions.

For digestion, the protein solution was reduced with 5 mM dithiothreitol for 30 min at 56 °C and alkylated with 11 mM iodoacetamide for 15 min at room temperature in darkness. The protein sample was then diluted by adding 100 mM TEAB to urea concentration less than 2M. Finally, trypsin was added at 1:50 trypsin-to-protein mass ratio for the first digestion overnight and 1:100 trypsin-to-protein mass ratio for a second 4 h-digestion.

The tryptic peptides were dissolved in 0.1% formic acid (solvent A), directly loaded onto a home-made reversed-phase analytical column (15-cm length, 75 μm i.d.). The gradient was comprised of an increase from 6% to 23% solvent B (0.1% formic acid in 98% acetonitrile) over 26 min, 23% to 35% in 8 min and climbing to 80% in 3 min then holding at 80% for the last 3 min, all at a constant flow rate of 400 nL/min on an EASY-nLC 1000 UPLC system. The peptides were subjected to NSI source followed by tandem mass spectrometry (MS/MS) in Q ExactiveTM Plus (Thermo) coupled online to the UPLC. The electrospray voltage applied was 2.0 kV. The m/z scan range was 350 to 1800 for full scan, and intact peptides were detected in the Orbitrap at a resolution of 70,000. Peptides were then selected for MS/MS using NCE setting as 28 and the fragments were detected in the Orbitrap at a resolution of 17,500. A data-dependent procedure that alternated between one MS scan followed by 20 MS/MS scans with 15.0s dynamic exclusion. Automatic gain control (AGC) was set at 5E4. Fixed first mass was set as 100 m/z.

The resulting MS/MS data were processed using Maxquant search engine (v.1.5.2.8). Tandem mass spectra were searched against human uniprot database concatenated with reverse decoy database. Trypsin/P was specified as cleavage enzyme allowing up to 4 missing cleavages. The mass tolerance for precursor ions was set as 20 ppm in First search and 5 ppm in Main search, and the mass tolerance for fragment ions was set as 0.02 Da. Carbamidomethyl on Cys was specified as fixed modification and acetylation modification and oxidation on Met were specified as variable modifications. FDR was adjusted to < 1% and minimum score for modified peptides was set > 40.

**1.17** | **Statistical analysis**

All results are presented as the mean ± SEM or SD as indicated. Statistical analysis was carried out using Student’s t test (two-tailed unpaired) for two groups, ANOVA for multi-group comparison, and the Kaplan-Meier method for mouse survival as indicated, all using GraphPad Prism 10 unless otherwise noted. Metabolomics and proteomics joint analysis was performed with MetaboAnalyst 5.0 ^29^. Gene Ontology analysis was performed in Gene Ontology PANTHER. Differences were considered significant when *P*<0.05.


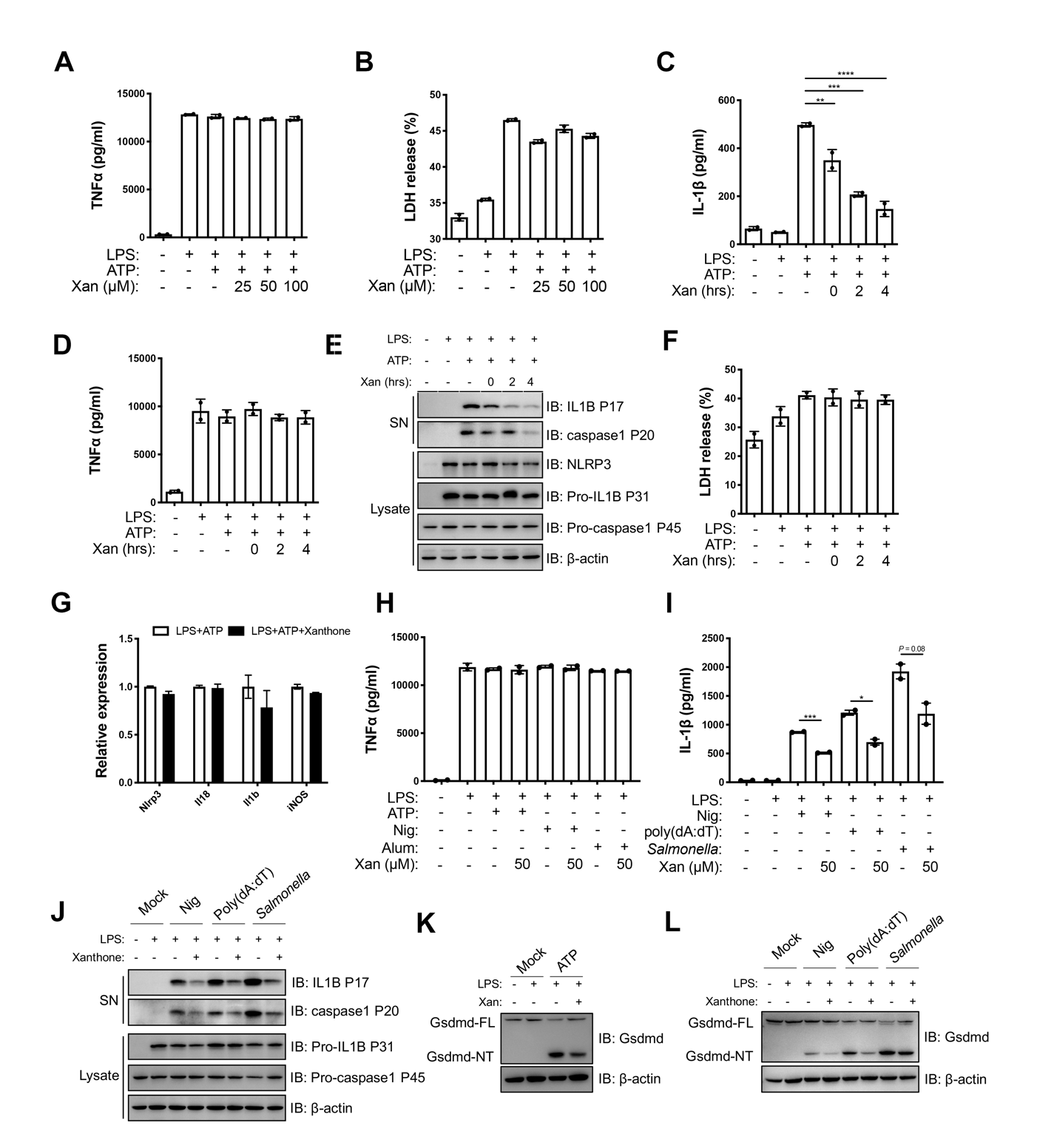


**Figure S1. Xanthone dose-dependently inhibits NLRP3 inflammasome activation with no effect on priming step.** (A and B) LPS-primed peritoneal macrophages treated with different doses of xanthone 2 hours before ATP challenge. TNFα (A) secretion was determined by ELISA, and LDH release was determined (B). (C-F) LPS-primed peritoneal macrophages treated with 50 µM xanthone for indicated time before ATP challenge. Supernatants (SN) and cell extracts (Lysate) were analyzed by immunoblotting (E), IL-1β (C) and TNFα (D) secretion was determined by ELISA. And LDH release was determined (F). (G) Relative expression of indicated genes were determined by RT-qPCR. (H) LPS-primed peritoneal macrophages treated with 50 µM xanthone for 2 hours, followed by stimulation with ATP, Nigericin (N), aluminum salts (Alum). Supernatants (SN) and cell extracts. Supernatants were also analyzed by ELISA for TNFα release. (I-L) LPS-primed peritoneal macrophages treated with 50µM xanthone before different inflammasome stimuli challenge. Supernatants (SN) and cell extracts (Lysate) were analyzed by immunoblotting (J-L) and IL-1β (I) secretion was determined by ELISA. **P* <0.05, ***P* < 0.01, ****P* < 0.001, *****P* <0.0001, two-tailed unpaired Student’s t-test.

**
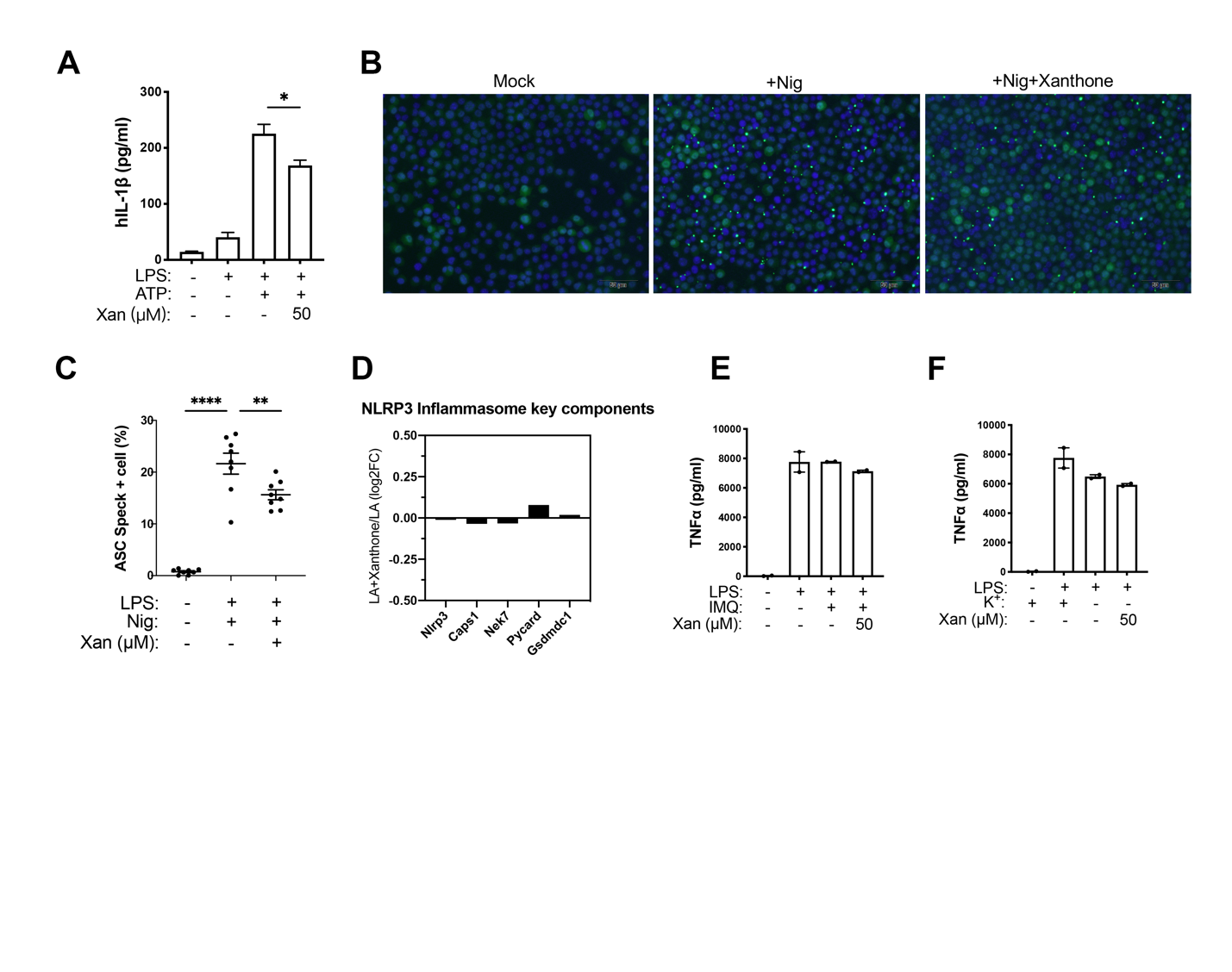
**

**Figure S2. Xanthone inhibits NLRP3 inflammasome assembly.** (A) LPS-primed PBMCs treated with 50 µM xanthone before ATP challenge as indicated. IL-1β secretion was determined by ELISA. (B and C) Representative immunofluorescence images of ASC speck formation in ASC-GFP-iBMDMs are shown in (B), and ASC speck positive cell percentage was calculated (C). Scale bars, 50 µm. (D) The protein levels of NLRP3 inflammasome components were determined by proteomics. (E and F) LPS-primed peritoneal macrophages treated with 50 µM xanthone before 100 µM Imiquimod challenge (E) or substitution of K^+^-free medium challenge for 30 min (F). TNFα secretion was determined by ELISA. ***P* <0.01, *****P* <0.0001, two-tailed unpaired Student’s t-test. Data are the mean ± SD.


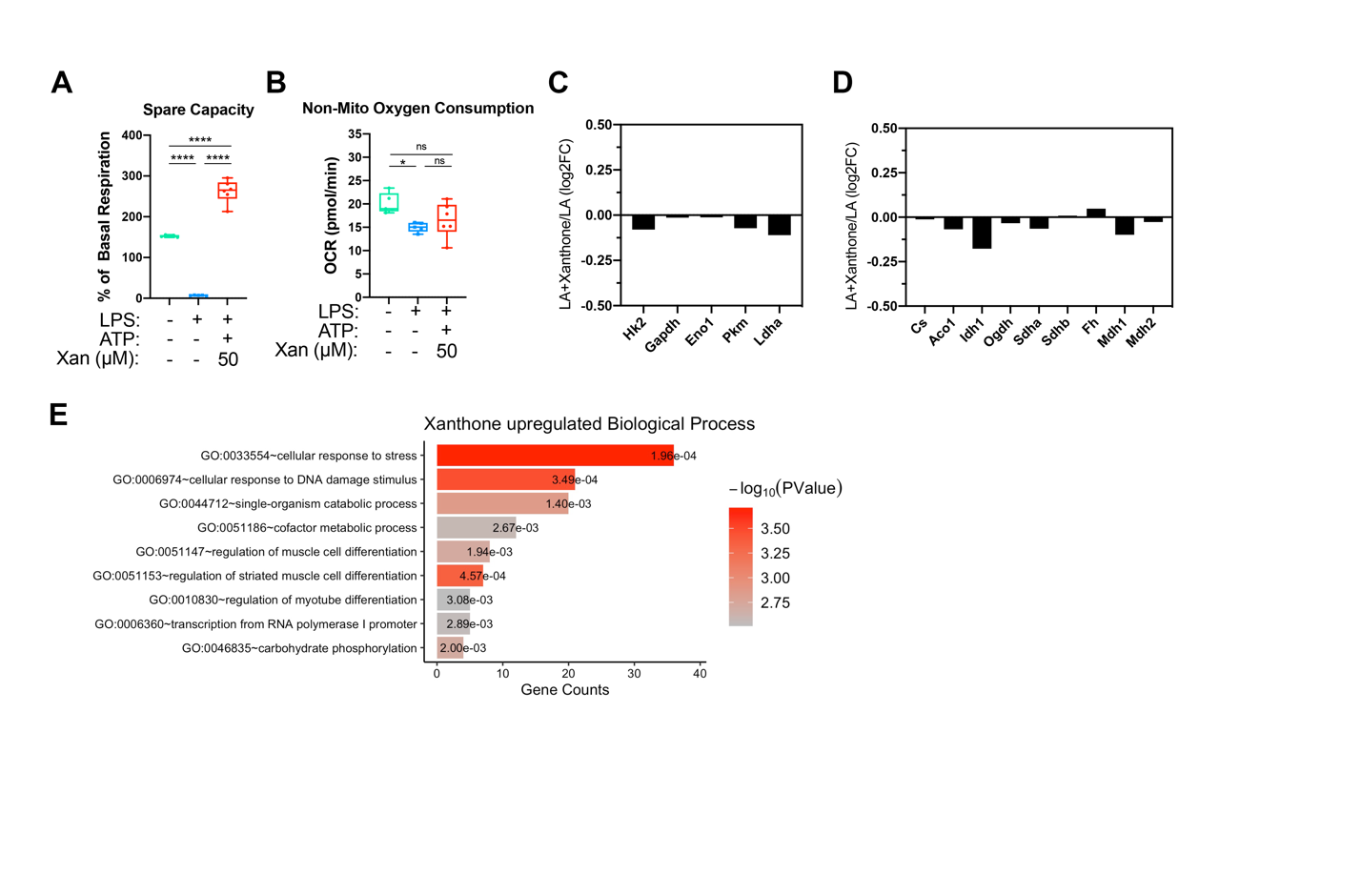


**Figure S3. Xanthone metabolically rewires macrophages to regain mitochondrial fitness upon NLRP3 inflammasome activation.** (A and B) Spare respiration and cell non-mitochondrial oxygen consumption determined based on oxygen consumption rate (OCR). (C and D) Fold changes of TCA cycle (C) and glycolysis (D) enzymes protein levels of TCA enzymes after xanthone treatment compared with control macrophages. (E) GO biological process enrichment of xanthone up-regulated proteins. **P* <0.05, *****P* <0.0001, one-way ANOVA.


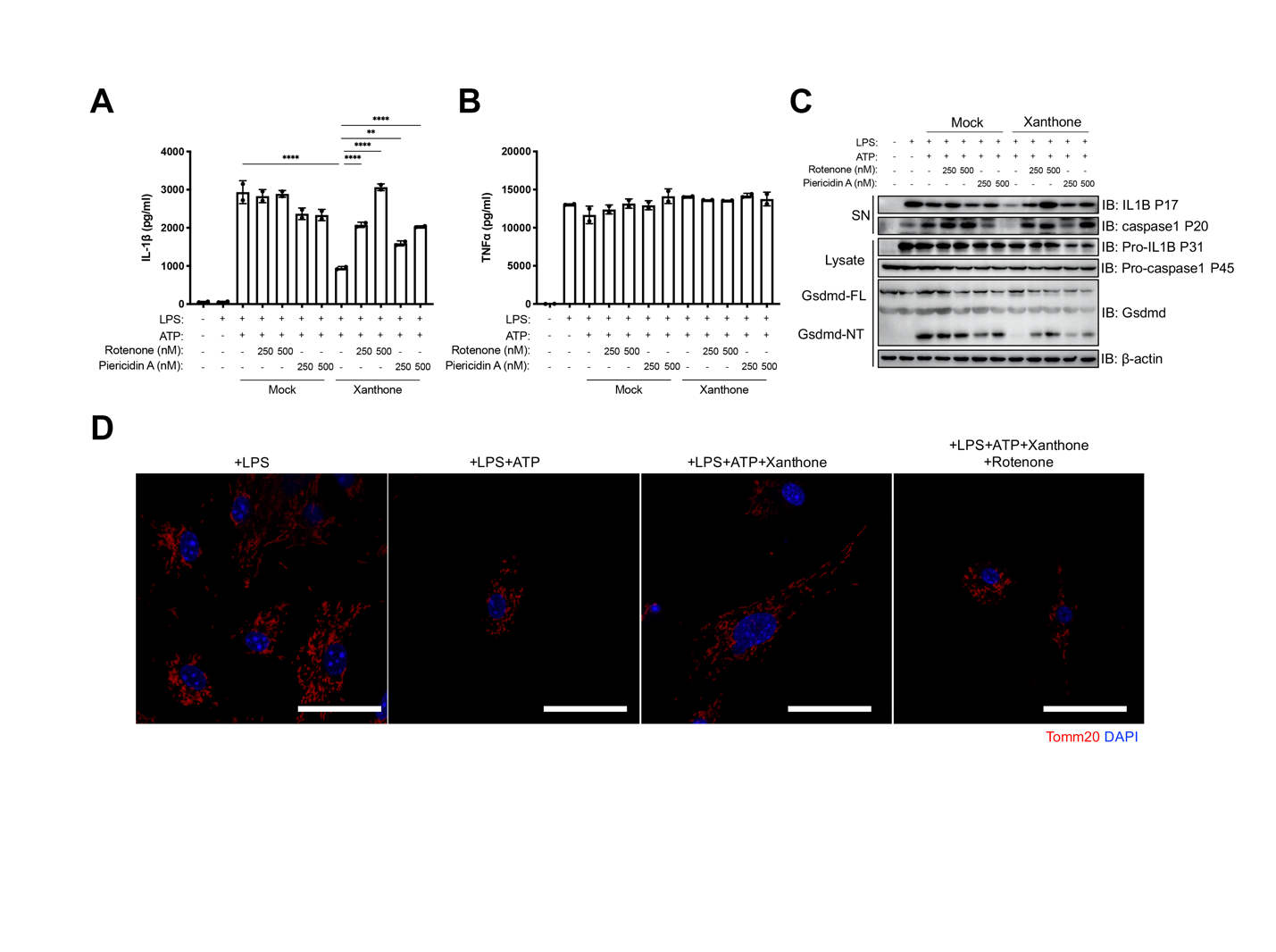


**Figure S4. Rotenone could rescue xanthone’s inhibition of NLRP3 inflammasome activation.** (A-C) LPS-primed peritoneal macrophages treated with 50 mM xanthone before ATP challenge. Cells were treated with rotenone and piericidin A with indicated concentration. Supernatants (SN) and cell extracts (Lysate) were analyzed by immunoblotting (C), IL-1β (A) and TNFα (B) secretion was determined by ELISA. (D) Representative confocal images of LPS-primed BMDMs pretreated with xanthone or not after ATP stimulation and 500nM rotenone for 30 min. Scale bars, 20 µm. ***P* <0.01, *****P* <0.0001, two-tailed unpaired Student’s t-test.


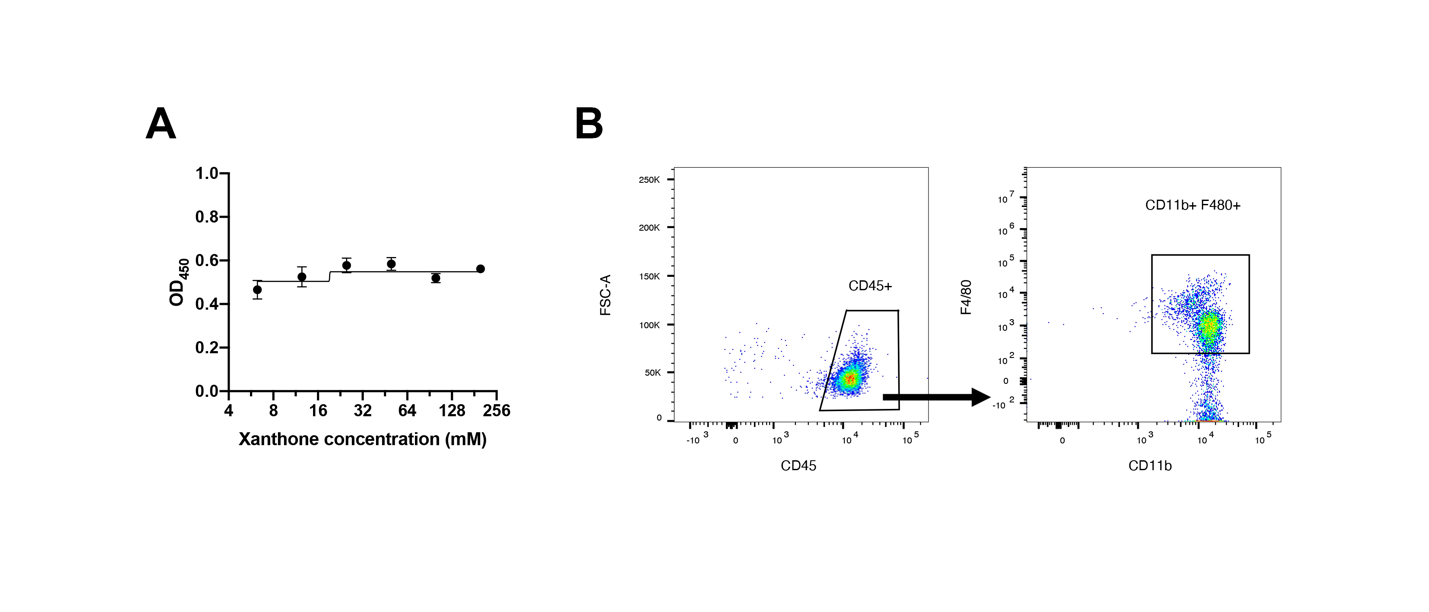


**Figure S5. Xanthone treatment improves mice keratitis.** (A) CCK8 assay of different doses of xanthone on human cornea epithelial cells. (B) Gating strategy of corneal infiltrating macrophages in Figures 4I and 4J.
